# Leaf Age and Position Effects on Quantum Yield and Photosynthetic Capacity in Hemp Crowns

**DOI:** 10.3390/plants9020271

**Published:** 2020-02-19

**Authors:** William L. Bauerle, Cole McCullough, Megan Iversen, Michael Hazlett

**Affiliations:** Department of Horticulture and Landscape Architecture, Colorado State University, Fort Collins, CO 80523, USA; cole.mccullough@rams.colostate.edu (C.M.); miversen@rams.colostate.edu (M.I.); mike.hazlett@rams.colostate.edu (M.H.)

**Keywords:** leaf longevity, leaf position, photosynthetic capacity, self-shading

## Abstract

We examined the aging of leaves prior to abscission and the consequences for estimating whole-crown primary production in *Cannabis sativa* L. (hemp). Leaves at three vertical positions in hemp crowns were examined from initial full leaf expansion until 42 days later. Photosynthetic capacity decreased as leaves aged regardless of crown position, light intensity, or photoperiod. Although leaves remained green, the photosynthetic capacity declined logarithmically to values of 50% and 25% of the maximum 9 and 25 days later, respectively. Plants grown under +450 μmol m^−2^ s^−1^ supplemental photosynthetically active radiation or enriched diffuse light responded similarly; there was no evidence that photoperiod or enriched diffuse light modified the gas exchange pattern. At approximately 14 days after full leaf expansion, leaf light levels >500 μmol m^−2^ s^−1^ decreased photosynthesis, which resulted in ≥10% lower maximum electron transport rate at ≥ 20 days of growth period. Furthermore, leaves were saturated at lower light levels as leaf age progressed (≤500 μmol m^−2^ s^−1^). Incorporating leaf age corrections of photosynthetic physiology is needed when estimating hemp primary production.

## 1. Introduction

Uniformity in leaf distribution throughout a canopy minimizes the spatial variability of the within^−^canopy solar radiation environment. Three-dimensional crown growth and development, however, changes the within-canopy architecture and light environment as new leaves develop and self-shade one another [1]. As canopies become denser, crown-to-crown competition among neighboring plants further increases the spatial and temporal crown light interception asymmetry [2,3]. These heterogeneity effects on the within-canopy light environment can accelerate leaf aging and subsequently decrease photosynthetic performance [3,4,5,6]. As a result, the distinction between age-related physiological deterioration versus light-gradient-related physiological acclimation to lower light intensities within a canopy is not always clear. Nonetheless, each are important for understanding the carbon budget of whole crowns.

Leaves develop from cell proliferation at apical meristems. As stem growth progresses, a natural leaf age chronosequence develops, followed by variations in photosynthetic capacity and efficiency [7]. Deciphering the effects of leaf longevity on photosynthetic capacity and efficiency within a crown is complicated by environmental factors (e.g., light acclimation) as well as sink effects that can correlate with decreases in photosynthetic productivity. Consequently, to evaluate the quantitative importance of optimal leaf longevity on whole-crown primary production, the contributions of leaves throughout the crown have to be considered (e.g., [3,6,8]).

Relative to the leaf development chronosequence of a plant crown, hemp stems develop leaves symmetrically along a space–time continuum (isotropic growth). For that reason, an actively growing non-stressed vegetative hemp crown provides a good system for investigating leaf age effects because it can be described by a single crown scalar that encompasses three-dimensional crown growth and development (e.g., [9]). Hemp crown growth and development thus provide a straightforward model system to determine the impact of leaf age and position effects on long-term carbon gain in canopies. Repeated measurements on the same leaves and leaves across various ages and crown positions are the most common means to investigate a photosynthetic capacity versus leaf longevity relationship [7]. 

In the absence of environmental stress, the quantum yield of photosynthesis (ϕCO_2_), or moles of photons absorbed that are converted to moles of CO_2_ fixed, is similar among C_3_ species [10,11,12]. For example, a ϕCO_2_ mean value of 0.093 was reported among widely divergent plant species [12]. Conversely, environmental stress such as oversaturating light can result in photoinhibition and subsequent reductions in photosynthetic efficiency and capacity [13]. To encompass the effects of leaf age, supraoptimal light, and extended photoperiod on photosynthetic function, we considered all three throughout the crown. Such leaf photosynthetic parameter information would be needed in leaf models estimating continuous seasonal fluctuations in carbon uptake.

We investigated the rate of decline of ϕCO_2_ and photosynthetic capacity for hemp leaves well in advance of abscission. The aim of this study was to investigate the changes in leaf gas exchange throughout the vegetative stage in *Cannabis sativa* ‘cherry wine’ crowns at three crown positions. We hypothesized that the age of a leaf from the time of full expansion could be used to predict ϕCO_2_ and photosynthetic capacity characteristics of hemp leaves when additional environmental stresses are absent (e.g., light availability, substrate nutrition, and water resources). If confirmed, leaf age after full expansion could be developed into a metric to inform canopy management decisions. For example, removal of older leaves throughout the crop cycle may enhance canopy CO_2_ uptake and ultimately crop yield. Thus, we characterized the age-related decline in ϕCO_2_ and photosynthetic capacity and developed leaf-age-based prediction models that will be used in a later paper to construct a carbon assimilation and utilization budget for hemp crowns.

## 2. Results

**Figure 1:**
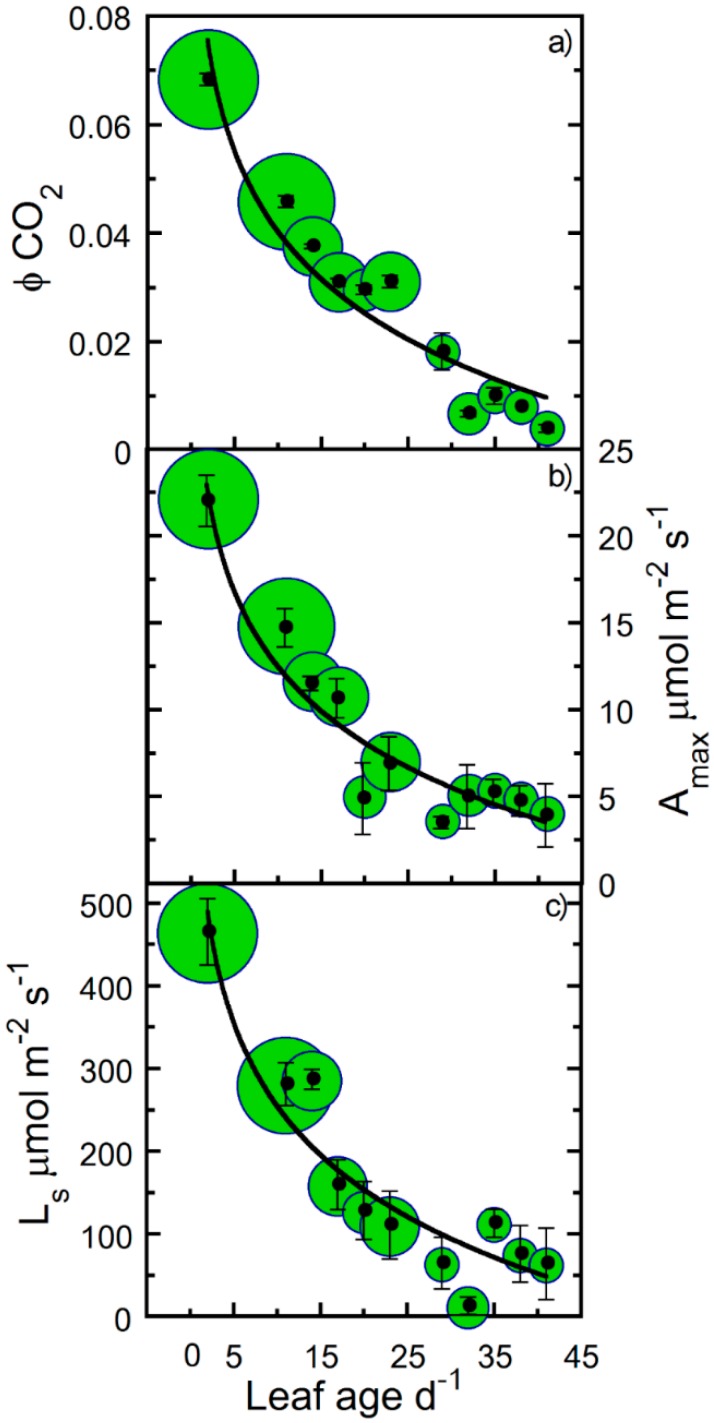
The quantum yield (ϕCO_2_), maximum photosynthesis (A_max_), and leaf light saturation (L_s_) of hemp leaves as a function of leaf age. Binned averages per three-day collection sequence (means ± SE) of measured changes in (**a**) ϕCO_2_, (**b**) A_max_, and (**c**) L_s_ versus leaf age. Samples were pooled for treatments and crown position, and sample size in bins is indicated by bubble diameter (n = 3–18 for ϕCO_2_, A_max_, and L_s_). Solid lines are logarithmic regression curves fitted to the entire data set.

**Figure 2:**
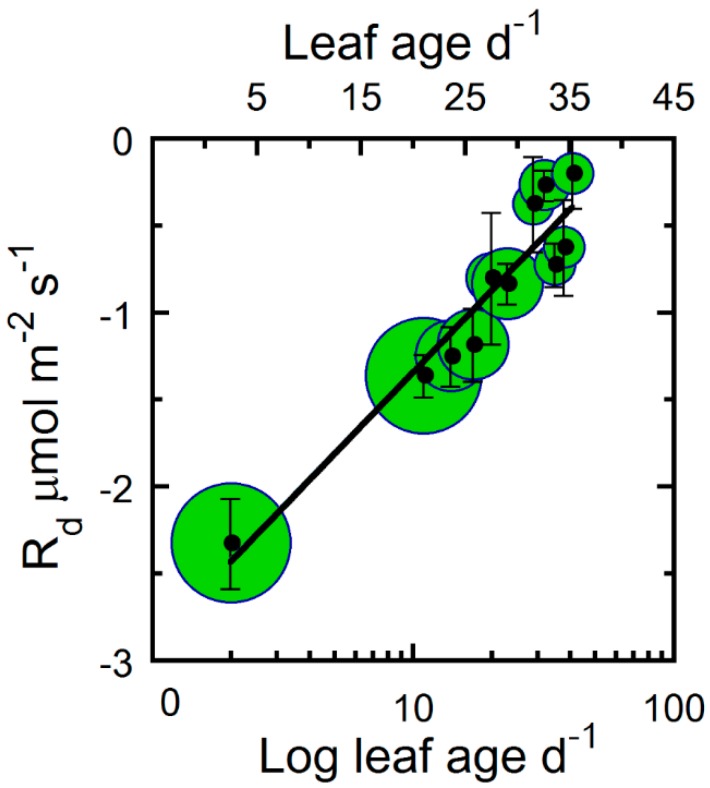
The leaf respiration rate (R_d_) of hemp leaves as a function of leaf age. The log base of leaf age and leaf age (primary and secondary *x*-axis, respectively). Binned averages per three-day collection sequence (means ± SE), where samples were pooled for treatments and crown position, and sample size in bins is indicated by the bubble diameter (n = 3–18 for R_d_). Solid line is a linear regression fitted to the entire data set.

**Figure 3:**
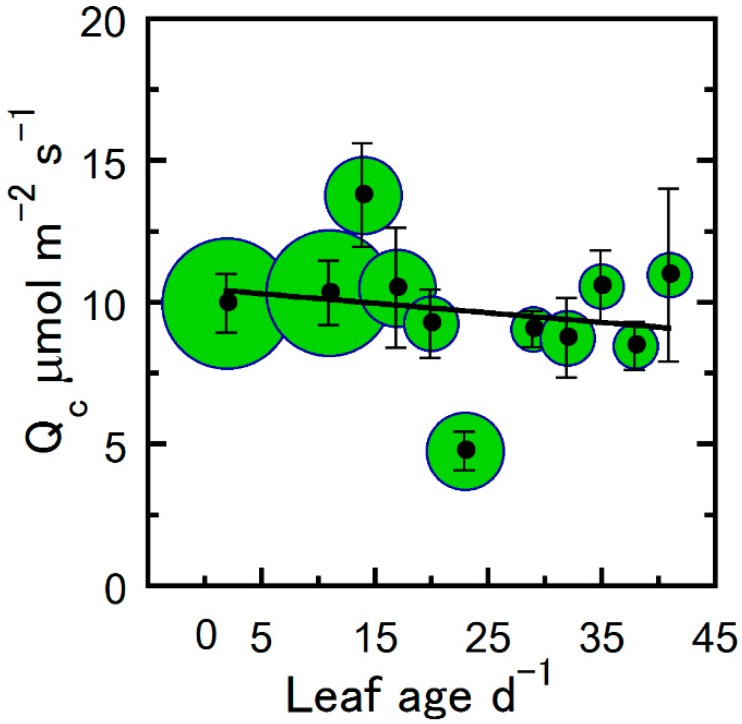
The light compensation point (Q_c_) of hemp leaves as a function of leaf age. Cuvette O_2_ was atmospheric ambient (~21%) and temperature was controlled at 25 °C. Binned averages per three-day collection sequence (means ± SE), where samples were pooled for treatments and crown position, and sample size in bins is indicated by the bubble diameter (n = 3–18 for Q_c_). Solid line is a linear regression fitted to the entire data set.

Figure 1 shows the decrease in maximum net photosynthesis (A_max_), ϕCO_2_, and leaf light saturation (L_s_) with leaf age for hemp. For all leaves, the highest photosynthetic activity was observed immediately after full leaf expansion and showed a steady decline as leaf age progressed (Figure 1). Compared to a C_3_ ϕCO_2_ mean of 0.093, the estimated hemp maximum ϕCO_2_ was comparable (logarithmic estimated *y*-intercept of ~0.091; Figure 1a). Using ϕCO_2_ as a biological reference, we therefore fitted Equation (3) to photosynthetic parameters A_max_, L_s_, maximum electron transport rate (J_max_), maximum Rubisco carboxylation rate (V_cmax_), CO_2_ compensation point (Γ_c_), and triose phosphate utilization (TPU). In addition, a decline in leaf dark respiration (R_d_) was observed; however, the R_d_ relationship with leaf age was best described by a simple log-transformed linear function (Figure 2). In contrast, the light response compensation point (Q_c_) stayed comparatively constant throughout the study period (Figure 3). For A_max_, ϕCO_2_, and L_s_, we observed a 10%–20% greater estimated maximum value compared with the observed maximum mean value (estimated *y*-intercept, Figure 1). Compared with the maximum estimated *y*-intercept value, A_max_ and R_d_ decreased by 50% and 75% after approximately 9 and 28 days (Figure 1 and Figure 2). Similarly, L_s_ and ϕCO_2_ decreased by 50% and 75% after approximately 8 and 22 days. Although photosynthetic capacity declined steadily after leaf full expansion, we note that the compensatory metabolic activity of R_d_ decreased.

**Figure 4:**
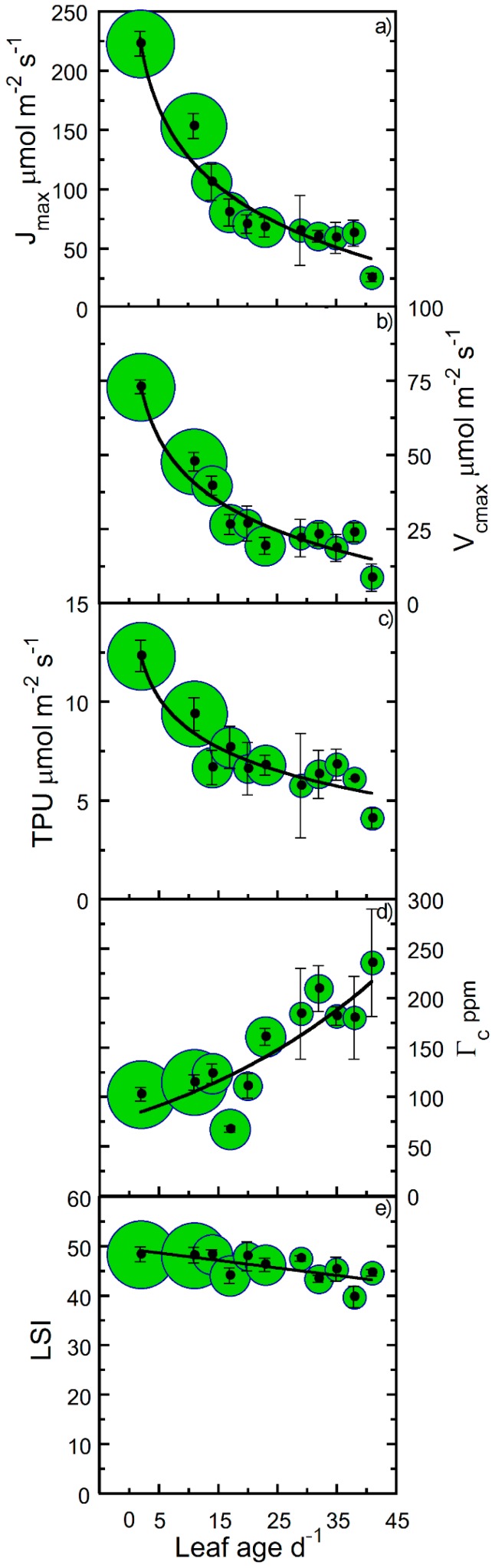
Maximum electron transport rate (J_max_), maximum Rubisco carboxylation rate (V_cmax_), triose phosphate utilization (TPU), CO_2_ compensation point (Γ_c_), and leaf spectral index (LSI) of hemp leaves as a function of leaf age. Binned averages per three-day collection sequence (means ± SE), where samples were pooled for treatments and crown position. Measured changes in (**a**) J_max_, (**b**) V_cmax_, (**c**) TPU, (**d**) Γ_c_, and (**e**) LSI (a proxy for leaf nitrogen content, chlorophyll a + b concentration, and greenness characteristics). Sample size in bins indicated by bubble diameter (n = 3–18 for J_max_, V_cmax_, TPU, Γ_c_, and LSI). Solid lines are logarithmic (panels a–d) and linear (panel e) regressions fitted to the entire data set.

V_cmax_, J_max_, Γ_c_, and TPU also decreased as leaf age progressed (Figure 4a–d). Table 1 shows the significance of correlations among variables that were expected to vary with leaf age for the pooled data. Leaf spectral index (LSI), an indicator of leaf chlorophyll and nitrogen per unit leaf area, did not show a significant change with leaf age (Table 1). For the majority of the study, LSI readings remained high and constant after full leaf expansion. Even though other photosynthetic parameters declined precipitously, LSI showed only a small decline (~6%) over the course of 42 days (Figure 4e). We note, however, that although LSI did not deteriorate appreciably during the study period, LSI values decreased abruptly 7–10 days after physiological measurements commenced (~50 days after leaf full expansion). For V_cmax_, J_max_, Γ_c_, and TPU, we observed a 10%–20% greater estimated maximum value compared with the observed maximum value (cf. *y*-intercept and maximum measured mean, Figure 4). In comparison with the estimated maximum *y*-intercept value, V_cmax_ and J_max_ decreased by 50% and 75% after approximately 9 and 28 days (Figure 4a,b). Similarly, Γ_c_ increased and TPU decreased by 50% after approximately 19 and 21 days (Figure 4c,d). 

**Figure 5:**
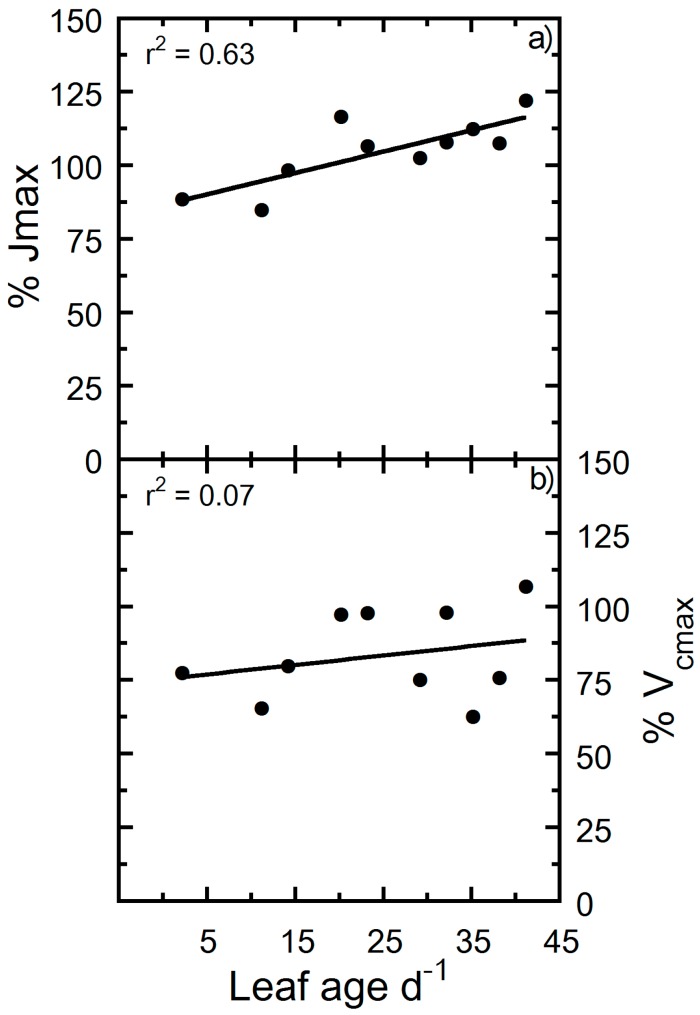
Percent change of photosynthetic physiology to photon flux of 1500 versus 500 μmol m^−2^ s^−1^. (**a**) Percent change of 500 relative to 1500 μmol m^−2^ s^−1^ maximum electron transport rate (J_max_) and (**b**) percent change of 500 relative to 1500 μmol m^−2^ s^−1^ maximum Rubisco carboxylation rate (V_cmax_). Solid lines are linear regressions fitted to the entire data set.

It is probable that high light intensity lowers values of V_cmax_ and J_max_. For example, the excess illumination at a light intensity of 1500 µmol photons m^−2^ s^−1^ versus 500 µmol photons m^−2^ s^−1^ could induce photoinhibition. To evaluate supraoptimal light effects on our parameter estimates, we performed net photosynthesis (A_n_) versus [CO_2_] (A_n_/C_i_) analysis on leaves under two disparate light intensities (500 versus 1500 µmol photons m^−2^ s^−1^), representing light intensities separated by approximately 50% of the sun’s intensity at the earth’s surface. Although both light intensities were either close to (500 µmol photons m^−2^ s^−1^) or in excess (1500 µmol photons m^−2^ s^−1^) of hemp’s L_s_ (Figure 1c), we showed that approximately 1000 µmol photons m^−2^ s^−1^ of additional light did not result in appreciable decreases in V_cmax_ and J_max_ for leaves younger than 14 days old (Figure 5). After a leaf age ≥20 days, an approximate 10% or greater decrease in J_max_ occurred at 1500 versus 500 µmol photons m^−2^ s^−1^ (Figure 5a). In contrast, V_cmax_ did not show signs of a decrease due to excess irradiance (Figure 5b). We noted a slightly lower V_cmax_ and J_max_ was observed at 500 versus 1500 µmol photons m^−2^ s^−1^ up to a leaf age of ~20 days. 

**Figure 6:**
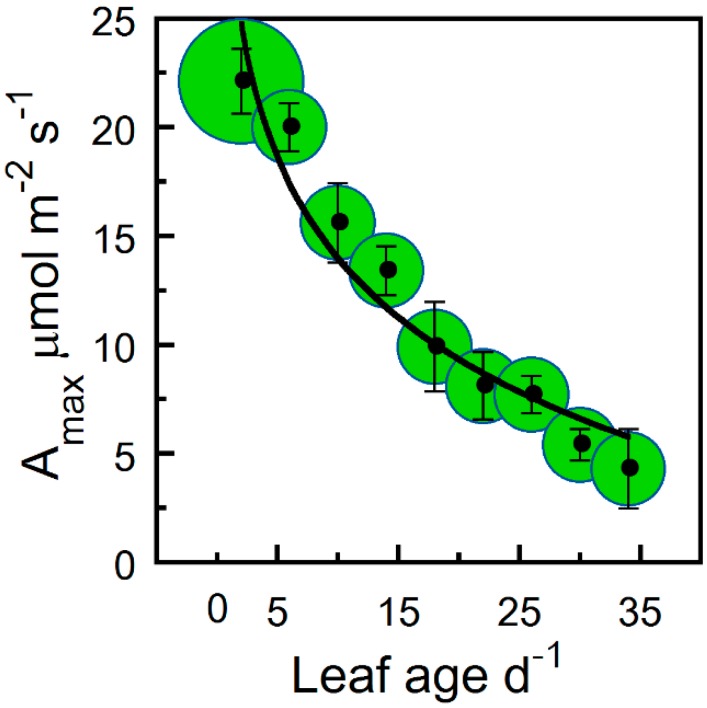
Maximum photosynthesis (A_max_) at crown layer 3 (the lowest crown position) of hemp leaves as a function of leaf age. Binned averages per three-day collection sequence (means ± SE), where samples were pooled for treatments and crown position. Sample size in bins indicated by bubble diameter (n = 3–18 for A_max_). Solid line is a logarithmic regression curve fitted to the leaf age data set.

Apart from our meristem measurements not encompassing the very earliest leaf growth stage due to inadequate leaf expansion for the gas exchange chamber, a decline in physiological activity was generally observed among A_max_, ϕCO_2_, V_cmax_, R_d_, J_max_, and TPU photosynthetic parameters as leaves aged. In addition, leaves of various ages at crown layer 3 (the lowest crown position) showed a similar A_max_ response as those among crown positions and treatments (cf. Figure 1b and Figure 6). Other than the linear log-transformed correlation of leaf age with R_d_ (Figure 2) and the relatively constant LSI and Q_c_, a logarithmic nontransformed relationship best characterized the correlation between the photosynthetic variable and leaf age (r = 0.66–0.96; Table 1). Table 2 shows the number of days after full leaf expansion for a decrease or increase (−, +) of 50% and 75% from the maximum mean physiological parameter value. Table 3 illustrates the logarithmic regression equations for the rate of change over time between leaf age and the physiological parameters. 

## 3. Discussion

The present study investigated the effect of leaf age on the gas exchange of hemp in order to understand the influences on leaf photosynthesis and underlying physiological traits. After the age of full leaf expansion, the photosynthetic capacity of leaves under different light treatments showed similar decreasing patterns of gas exchange in relation to their age (cf. Figure 1 and Figure 3). There was no evidence that photoperiod or enriched diffuse light modified the gas exchange pattern. This indicates that younger leaves have a higher capacity to accumulate photosynthate [3]. The proportion of young leaves in high light is therefore of critical importance to canopy photosynthesis [14].

Other gas exchange experiments using data collected from leaves at various ages have also implicated leaf age as a major contributing factor to photosynthetic capacity. Our results add to this growing evidence that photosynthetic capacity declines as leaves age after full expansion [5,15,16,17,18,19]. The age-related decrease in photosynthetic capacity may be partly caused by decreased mesophyll conductance and stomatal limitation [20,21], although the concurrent decrease in R_d_ along with photosynthetic capacity in hemp appears to support the hypothesis that leaf age directly affects leaf biochemical properties.

The data show evidence of young canopy leaves with a higher A_max_ that falls in conjunction with increased leaf age (Figure 6). We calculated how the A_n_/C_i_ parameters behaved under high light compared to those from curves generated at approximately 50% lower irradiance. We observed that in leaves ≥20 days old, J_max_ decreased approximately 10% when exposed to 1500 µmol photons m^−2^ s^−1^ as compared with 500 µmol photons m^−2^ s^−1^. This suggests that a simple downregulation function based on leaf age might serve as an easily measured index of leaf photosynthetic capacity. An index would have practical applications in research on hemp carbon fixation and be applicable in leaf-to-canopy upscaling schemes. For example, by mapping the distribution of leaf age throughout a crown, the whole-canopy performance may be compared among canopies comprised of different age leaves [22,23]. The challenge when modeling the time course of carbon fluxes would be to identify architectural properties that positively impact the total canopy CO_2_ uptake [24].

Identification of optimal canopy architectural features for potential photosynthetic improvements could focus breeding efforts of genetically modifiable leaf traits [25,26]. It could also be used to study the influence of canopy architecture on canopy photosynthesis. Although it may be convenient to use LSI as a proxy for chlorophyll in some species due to the ability to sense it via remote satellite reflectance, our results suggest this approach would not be accurate for tracking changes in photosynthetic capacity in well-fertilized hemp. Additional research is needed to determine specific changes in leaf N and chlorophyll relative to LSI if remote sensing is to be used to estimate changes in photosynthetic capacity and performance.

Measured responses of A_max_ for hemp leaves at various ages on the lowest crown position (the position that offered the largest lateral gradient in leaf ages) resulted in a similar leaf-age-related response as compared to the logarithmic rate of decrease that was observed across crown positions (cf. Figure 1b and Figure 6). Due to leaf age decreasing photosynthetic activity, we can discern strategies for dealing with the leaf age effects, for instance, restricting substantial levels of irradiance to the top layers by closing the canopy to protect the lower layers and to ensure a high degree of light saturation for the upper layers.

Increases in photosynthetic capacity are frequently observed in new leaves that are building physiological and structural capacity. Once leaves are fully expanded, photosynthetic capacity declines linearly with leaf age in many plant species despite favorable growing conditions [27,28]. We found a logarithmic function provided the best fit to our data, together with a physiological justification: the estimated ϕCO_2_ *y*-intercept of 0.091 was comparable to the C_3_ mean of 0.093 [12]. Consistent with some crown level studies, we found the effect of leaf age to vary temporally but not spatially within the crown, where decreases in A_max_, for example, have been found to be related to a leaf age reduction in V_cmax_ (e.g., [20,21]). When two variables are correlated, there may or may not be a causative connection, and this connection may be indirect. Our correlational results demonstrate that leaf age can predict photosynthetic capacity in hemp (Table 1). Studies to ascertain the underlying mechanism by which leaf age affects photosynthetic capacity, such as investigations of hemp cell half-life, should be a priority for future research.

In conclusion, we measured a progressive decline in V_cmax_ and J_max_ in hemp during the vegetative stage, supporting the need for age-related downscaling of these parameters. Overall, our results indicate that leaf age is an important temporal factor in hemp crowns. Our logarithmic modeling approach significantly simplifies the description of crown photosynthetic parameters as well as approximates the photosynthetic capacity of hemp leaves at various stages of ontogeny. From a canopy management view, our correlation models of leaf-age-related physiological deterioration simplify the description of age effects on hemp photosynthetic activity by permitting temporal scalars to modify photosynthetic parameters when predicting hemp crown productivity. This makes it possible to extract canopy gas exchange estimates that would otherwise be expensive and labor intensive to measure in the field. The next step is to incorporate leaf model parameterizations into a canopy-scale gas exchange model to effectively extrapolate the leaf-scale results to a whole canopy. 

## 4. Materials and Methods 

A preliminary study was conducted solely to understand hemp crown development and morphology at the Colorado State University Horticulture Center in Fort Collins, CO, USA. Thirty seed-based, two-week-old plugs of *Cannabis sativa* ‘cherry wine’ were transplanted into 11 L bato buckets filled with horticulture perlite. Treatments consisted of two independent 1.5 × 2.5 m plots of 15 equally spaced plants: one under natural light and the other under natural light plus an extended photoperiod with supplemental light (+450 μmol m^−2^ s^−1^ photosynthetic photon flux (PPF) via high-pressure sodium lamps (600 W Xtrasun, Hydrofarm Inc. Petaluma, CA, USA)). To ensure adequate nutrients and water, Peters’ Excel Cal-Mag (15-5-15) was applied twice daily at a strength of 200 ppm N through microemitter irrigation (360° substrate surface drip rings). All plants were allowed to acclimate for 14 days prior to crown morphology sampling.

We sampled total plant height (HT), stem diameter (SD), stem length, and three-dimensional crown size (*x*, *y*, and *z* directions, in meters) on all plants six times over the course of the growing cycle to assess isotropic growth characteristics. Immediately after each of the six morphology measurement intervals, leaf area was estimated by a destructive harvest on a randomly selected plant per treatment. The crowns were equally subdivided into three vertical sections and all leaves per sector were removed and measured for total area (LiCor 3100, Lincoln, NE, USA). Fresh and dry weights were determined separately for leaves and stems, and dry mass was determined after being oven-dried at 70 °C for approximately 3–5 days.

After the preliminary study, which allowed us to gain an understanding of the chronosequence of leaf development throughout a hemp crown, we conducted leaf gas exchange measurements on new and independent plant material from 7 August 2019 to 1 October 2019 at the Colorado State University Horticulture Center. Sixty feminized seeds of *Cannabis sativa* ‘cherry wine’ were sown on 11 July 2019 and transplanted into 5 × 7.5 cm rock wool cubes on 25 July 2019. On 31 July 2019, plants were transplanted into 11 L bato buckets and growing methods were as described in the preliminary experiment. Thirty replicate plants were randomly transferred to either a natural (n = 15) or extended photoperiod with supplemental light (n = 15) treatment. The extended photoperiod treatment maintained day length at 18 h; photoperiod varied in the natural treatment as day length changed. An additional 30 plants were transferred into an enriched diffuse light environment where reflective white plastic covered the floor surface and crown–crown competition among neighboring plants was minimal (crown–crown spacing was >3× of the extended and natural photoperiod treatments). The diffuse light enrichment was approximately 150% at the lowermost crown periphery (height above ground level ~0.4 m) (LiCor 189, 191R, Lincoln, NE, USA) and the photoperiod varied as in the natural treatment.

### 4.1. Leaf Gas Exchange and Light Absorption Measurements

To examine the age-related photosynthetic capacity characteristics, three replicate plants per treatment located in the center of the plot were sampled for changes in leaf gas exchange traits. The south-facing second node branchlet leaf of the fifth main stem node from the substrate surface was tagged and repeatedly measured. Initially, the crown position encompassed the topmost fully expanded sunlit leaves and leaf age was defined as the days immediately after full leaf expansion. Due to isometric indeterminate growth during the vegetative phase of the growth cycle, the 10th and 15th node branchlets were added once the crown developed fully expanded leaves at the respective crown positions; that is, the crown was ultimately divided into three strata (the upper, middle, and bottom third of the live crown). Due to successive leaf production in hemp, this sampling regime facilitated the sequential addition of the middle and bottom crown positions at approximately 3 and 6 weeks into the study, together with repeated measurements of the same individual leaves. Periodically, measurements of leaves with contrasting ages and positions within a branch took place on a given sampling day. Gas exchange was repeatedly measured at each canopy position on fully expanded leaves of three replicate plants per treatment using a portable gas exchange system (CIRAS-2, PP Systems, Haverhill, MA, USA) fitted with a light- and environmentally controlled cuvette (Model PLC (U), PP Systems). The measurements were performed at a controlled leaf temperature of 25 °C and vapor pressure deficit (VPD) of 1.2 kPa.

Response curves of net photosynthesis (A_n_) versus [CO_2_] (A_n_/C_i_) and photosynthetically active radiation (Q_p_) (A_n_/Q_p_) were repeatedly run on three plants per treatment at three canopy positions. For the A_n_/C_i_ curves, leaves were acclimated in the chamber for 5–10 min until A_n_ was stable at a controlled CO_2_ concentration (415 μmol mol^−1^) and light level (1500 μmol m^−2^ s^−1^). The CO_2_ response curve was constructed at CO_2_ levels of 415, 300, 200, 150, 100, 50, 25, 450, 500, 800, 1000, 1500, and 415 μmol CO_2_ mol^−1^. After the completion of the A_n_/C_i_ curve, CO_2_ concentration was kept constant at 415 μmol mol^−1^ and Q_p_ was sequentially lowered from 1500 to 1200, 1000, 800, 600, 400, 200, 175, 150, 125, 100, 80, 60, 40, 25, 20, and 0 μmol m^−2^ s^−1^. Due to the time consumption of generating a A_n_/Q_p_ and A_n_/C_i_ curve per leaf and the continuous addition of new leaves as canopy positions developed, the younger leaf sample size ended up being larger than that of older leaves. Immediately after gas exchange, five replicate light absorptance samples were recorded at that leaf with a SPAD meter and averaged (model 502B, Minolta Inc., Ramsey, NJ, USA). The SPAD values were used to correct ϕ_a_ to ϕ as described in [29] and to track the changes in the LSI [30,31,32]. After the initial three measurement periods at node 5, we started to observe downregulation of leaf light saturation. Thus, measurement periods 4–11 subsequently added an additional A_n_/C_i_ response curve generated at 500 μmol m^−2^ s^−1^ Q_p_ to cover the effects of excess light on photosynthetic function.

### 4.2. Photosynthetically Active Radiation Measurements

Two line quantum sensors (model LI191R, Li-Cor Inc., Lincoln, NE, USA) were placed parallel to the north/south row orientation adjacent to the plant stems. A third line sensor was placed 0.5 m above the canopy. The line sensors sampled PAR every minute and then recorded a 15 min average (CR10x; Campbell Scientific, Logan, UT, USA).

### 4.3. A_n_/C_i_ Curve Fitting

The model of Farquhar et al. [33] was used to estimate the maximum Rubisco carboxylation rate (V_cmax_) and maximum rate of electron transport for RuBP regeneration (J_max_) under saturating light. The A_n_/C*_i_* data were analyzed as per the default fitting method “fitaci” function of the “plantecophys” package [34] according to Wullschelger [35]:

A_n_ = (1 − 0.5/τC*_i_*) × min (V_c_, W_j_, T_p_) − R_L_ (1)

where the minimum of any of the three factors—Rubisco activity (V_c_), RuBP regeneration (W_j_), and regeneration of inorganic phosphate (T_p_)—can limit CO_2_ assimilation. Tau (τ) represents the specificity factor for Rubisco [36]. Respiration in the light (R_L_), comprising mainly of processes other than photorespiration, refers to the release of CO_2_ in the light [37]. The CO_2_ compensation point (Γ_c_) was estimated from the intersection of the regression line with the *x*-axis. Equation (1) was fitted separately for A_n_/C*_i_* curves at 1500 and 500 μmol m^−2^ s^−1^.

### 4.4. A_n_/Q_p_ Curve Fitting

Photosynthesis versus Q_p_ data were fit to the nonrectangular hyperbola model of Parsons et al. [38] using least-squares regression:

A_n_ = ϕ_a_ Q_p_ + A_max_ − √(ϕ_a_ Q_p_ + A_max_)^2^ − 4 ϕ_a_ Q_p_ ø A_max_ /2 − R_d_ (2)

where A_max_ is the light-saturated net photosynthetic rate, ϕ_a_ is the apparent quantum yield of assimilation, ø is the convexity of the curve, and R_d_ is the respiration rate. The parameter ϕ_a_ was calculated by linear regression analysis on the initial slope from 20 to 125 μmol m^−2^ s^−1^ to exclude the Kok effect region (≤20 μmol m^−2^ s^−1^) [39] and prevent ϕ_a_ underestimation from data in the nonlinear region (≥125 μmol m^−2^ s^−1^) [40]. The light compensation point (Q_c_) was estimated from the intersection of the regression line with the *x*-axis (A_n_ = 0), and R_d_ was measured at the end of the A_n_/Q_p_ curve. Quantum yield for CO_2_ (ϕCO_2_) was derived from ϕ_a_ by correcting ϕ_a_ for the percentage leaf absorptance of Q_p_ as described in Bauerle et al. [29]. Equation (2) was fitted separately for the independent A_n_/Q_p_ curves.

### 4.5. Parameterizations of Leaf Photosynthetic Longevity

The functional form of the rate of change in A_max_, ϕCO_2_, L_s_, J_max_, V_cmax_, Γ_c_, and TPU with leaf age can be expressed in the following leaf-age-based logarithmic scalar function (e.g., ϕCO_2_):

ϕCO_2_ = *a*ln(L_a_) + b (3)

where *a* and *b* are the fit constants of the logarithmic (ln) least-squares regressions between the rate of change for A_max_, ϕCO_2_, L_s_, J_max_, V_cmax_, Γ_c_, and TPU and leaf age (L_a_), respectively. From a causal view, the form of Equation (3) introduces biological function by allowing each physiological parameter to achieve its theoretical maximum at the time of full leaf expansion. The model was validated for ϕCO_2_ via the known requirement of 8 mol of photons to reduce 1 mol of CO_2_ where the theoretical maximum ϕCO_2_ = 0.125. Due to cyclic photophosphorylation, 0.125 is then reduced to ~0.112 and further reduced to ~0.092 by means of CO_2_ released through photorespiration measured at atmospheric O_2_ levels [10,12,40]. A ϕCO_2_ of ~0.092 would then decrease further due to a deterioration in physiological function [12]. 

### 4.6. Statistical Analysis 

The gas exchange experiment was designed to allow us to discriminate between effects due to leaf age, effects due to crown position, and any subsequent interactions due to light environment (e.g., enriched diffuse light). The total sample size for A_n_/Q_p_ and A_n_/C_i_ curves was 27 leaves (n = 9 under ambient, n = 9 under +450 μmol m^−2^ s^−1^ PPF supplemental light, and n = 9 under enriched diffuse radiation). Of the nine leaves per treatment, n = 3 replicate leaves were at each of three crown positions per treatment. Leaves ranged in age from 1 to 42 days. The curve parameters were evaluated at three-day age interval ranges. A one-way ANOVA with the least significant difference at a significance level of *p* > 0.05 was used to assess photosynthetic parameters among treatment factors per three-day interval and was found to not be significantly different among treatments and crown positions. Samples were pooled among treatments and crown positions and subsequently binned to build a general leaf age effect equation per photosynthetic parameter. Logarithmic least-squares and linear regression was fitted between photosynthetic parameters and leaf age, treating age as a continuous variable. In addition, we investigated exponential, polynomial, and power correlation analysis to assess the strength of the coefficient of determination (*r*^2^). For leaf ages beyond day 17, there was not a balanced design at every measurement interval due to the time consumption of generating an A_n_/Q_p_ and two A_n_/C_i_ curves per leaf; so after day 17, we used a one-way ANOVA with replicate and treatment, nesting replicate within treatment.

## Figures and Tables

**Table 1:** Pearson’s correlation coefficients (*r*) and significance levels (*p*) for correlated variables. Samples were pooled for treatments and crown position. Leaf respiration rate (R_d_), quantum yield (ϕCO_2_), maximum photosynthesis (A_max_), leaf light saturation (L_s_), light compensation point (Q_c_), maximum electron transport rate (J_max_), maximum Rubisco carboxylation rate (V_cmax_), triose phosphate utilization (TPU), CO_2_ compensation point (Γ_c_), and leaf spectral index (LSI, a proxy for leaf nitrogen content, chlorophyll a + b concentration, and greenness characteristics) versus leaf age. Total number of leaves = 27.

Correlated Variable	*r*	*p*
R_d_ vs. Age	0.95	<0.00000
A_max_ vs. Age	0.96	<0.00000
L_s_ vs. Age	0.95	<0.00000
ϕCO_2_ vs. Age	0.94	<0.00000
Q_c_ vs. Age	0.04	0.60
LSI vs. Age	0.57	0.07
J_max_ vs. Age	0.96	<0.00000
V_cmax_ vs. Age	0.96	<0.00000
TPU vs. Age	0.93	0.00003
Γ_c_ vs. Age	0.66	0.03

**Table 2:** Days after full leaf expansion when an estimated decrease or increase (−, +) of 50% and 75% from the maximum mean physiological parameter value occurred. Samples were pooled for treatments and crown position, and estimates were calculated with either a linear (*) or logarithmic (†) equation. Leaf respiration rate (R_d_), quantum yield (ϕCO_2_), maximum photosynthesis (A_max_), leaf light saturation (L_s_), light compensation point (Q_c_), maximum electron transport rate (J_max_), maximum Rubisco carboxylation rate (V_cmax_), triose phosphate utilization (TPU), CO_2_ compensation point (Γ_c_), and leaf spectral index (LSI, a proxy for leaf nitrogen content, chlorophyll a + b concentration, and greenness characteristics) versus leaf age. Total number of leaves = 27.

Parameter	(−, +)	50%	75%	Equation Type
A_max_	−	9	25	†
L_s_	−	8	20	†
ϕCO_2_	−	8	22	†
R_d_	−	9	25	*
Q_c_	−	>100	>100	*
J_max_	−	9	27	†
V_cmax_	−	9	28	†
TPU	−	21	93	†
Γ_c_	+	19	24	†
LSI	−	>100	>100	*

**Table 3:** Rate of change over time logarithmic and/or linear regression equations between leaf age and physiological parameters. Samples were pooled for treatments and crown position and fitted with either a linear (*) or logarithmic (†) equation. Leaf respiration rate (R_d_), quantum yield (ϕCO_2_), maximum photosynthesis (A_max_), leaf light saturation (L_s_), light compensation point (Q_c_), maximum electron transport rate (J_max_), maximum Rubisco carboxylation rate (V_cmax_), triose phosphate utilization (TPU), CO_2_ compensation point (Γ_c_), and leaf spectral index (LSI, a proxy for leaf nitrogen content, chlorophyll a + b concentration, and greenness characteristics) versus leaf age. Total number of leaves = 27.

Parameter	*r* ^2^	Slope*ln(x)	*+y*-Intercept	Equation Type
R_d_	0.91	0.68 *	−2.898	*
A_max_	0.92	−6.419	27.471	†
L_s_	0.89	−145.9	588.31	†
ϕCO_2_	0.91	−0.022	0.091	†
Q_c_	0.04	−0.456 *	11.001	*
LSI	0.32	−0.152 *	49.334	*
J_max_	0.93	−61.47	269.8	†
V_cmax_	0.92	−19.65	87.833	†
TPU	0.93	−2.312	13.956	†
Γ_c_	0.63	3.546	67.292	†
